# Fifteen Challenges in Establishing a Multidisciplinary Research Program on eHealth Research in a University Setting: A Case Study

**DOI:** 10.2196/jmir.7310

**Published:** 2017-05-23

**Authors:** Helena Grönqvist, Erik Martin Gustaf Olsson, Birgitta Johansson, Claes Held, Jonas Sjöström, Annika Lindahl Norberg, Emma Hovén, Robbert Sanderman, Theo van Achterberg, Louise von Essen

**Affiliations:** ^1^ U-CARE Department of Women's and Children's Health Uppsala University Uppsala Sweden; ^2^ Department of Immunology, Genetics and Pathology Experimental and Clinical Oncology Uppsala University Uppsala Sweden; ^3^ Department of Medical Sciences Cardiology Uppsala University Uppsala Sweden; ^4^ Uppsala Clinical Research Center Uppsala Clinical Research Center Uppsala University Uppsala Sweden; ^5^ Department of Informatics and Media Campus Gotland Uppsala University Uppsala Sweden; ^6^ Centre for Occupational and Environmental Medicine Stockholm County Council Stockholm Sweden; ^7^ Department of Health Psychology University Medical Center Groningen University of Groningen Groningen Netherlands; ^8^ Department of Psychology, Health and Technology University of Twente Enschede Netherlands; ^9^ Academic Centre for Nursing and Midwifery Department of Public Health and Primary Care KU Leuven Leuven Belgium

**Keywords:** organization and administration, eHealth, interdisciplinary studies

## Abstract

**Background:**

U-CARE is a multidisciplinary eHealth research program that involves the disciplines of caring science, clinical psychology, health economics, information systems, and medical science. It was set up from scratch in a university setting in 2010, funded by a governmental initiative. While establishing the research program, many challenges were faced. Systematic documentation of experiences from establishing new research environments is scarce.

**Objective:**

The aim of this paper was to describe the challenges of establishing a publicly funded multidisciplinary eHealth research environment.

**Methods:**

Researchers involved in developing the research program U-CARE identified challenges in the formal documentation and by reflecting on their experience of developing the program. The authors discussed the content and organization of challenges into themes until consensus was reached.

**Results:**

The authors identified 15 major challenges, some general to establishing a new research environment and some specific for multidisciplinary eHealth programs. The challenges were organized into 6 themes: Organization, Communication, Implementation, Legislation, Software development, and Multidisciplinarity.

**Conclusions:**

Several challenges were faced during the development of the program and several accomplishments were made. By sharing our experience, we hope to help other research groups embarking on a similar journey to be prepared for some of the challenges they are likely to face on their way.

## Introduction

### Background

Publicly funded multidisciplinary eHealth research environments face challenges seldom described systematically in the literature [1]. This paper aims to start to fill this gap. In the following, we describe both foreseen and unforeseen challenges that arose when a relatively large research program studying eHealth solutions for people suffering emotionally with serious somatic illnesses was set up. Some of the challenges discussed below are general for creating research environments, whereas others are more specific for multidisciplinary eHealth projects.

Societies aim to support the research that best meets their needs at the time. In Sweden, based on a proposal from the government (proposition 2008/09:50), a plan to support large strategic research environments was launched from the main public research funding agencies. The overall aim was to strengthen Sweden’s position as a research nation and thereby increase its scientific competitiveness in a globalized world. It also explicitly promoted multidisciplinary research on eHealth. The Uppsala University Psychosocial Care Program (U-CARE) was one of the 43 programs that were funded for 5 years based on this proposition. Thereafter, the funding of this program has been prolonged by 1 year at a time. U-CARE was, and still is, a multidisciplinary program involving the academic disciplines of caring science, clinical psychology, health economics, information systems, and medical sciences. U-CARE’s main vision was, and still is, to increase cost-effective access to participatory mental health care in connection with somatic illness by using welfare technology. An online platform, the U-CARE-portal, was developed in house to support provision of self-care, care, and psychological treatment. It was designed to serve as a research backbone for the U-CARE program with built-in features such as stratified randomization, flexible data collection, logging of patient and therapist behaviors, automatic reminders, and providing an overview on study progress (eg, number of included and randomized participants).

The background for the governmental support for U-CARE was the rapid development in information and communication technology and the role of Internet, which has influenced how we get information and communicate about health. Interactive eHealth programs for behavioral change have gradually become more available and have attained research-based support of its efficacy in several areas [2]. However, there are also several new difficulties emerging as these new tools for health care become common. For example, eHealth solutions offered by public and commercial actors are now becoming so common and diverse that the quality is difficult to manage, and the shift from face-to-face to Web-based communication in health interventions may result in that even the appropriate research and evaluation methodology has to be reconsidered [3,4]. Implementation, legal matters, ethics, and integrity issues as well as technical and practical concerns are other areas where eHealth faces new and different challenges than the traditional health care does [5,6]. There is certainly a need for sustainable, multidisciplinary research environment in this area, as the potential benefits from using eHealth on a large scale for a society are many and large [7].

### U-CARE’s Project Goals

Five goals to be achieved by 2014 were specified in the original grant application for the U-CARE program. As written in the 2009 application, the goals were to (1) establish an internationally competitive, innovative psychosocial research platform that will be applied within pediatric oncology, adult oncology, and cardiology; (2) build a high-quality research-based, transdisciplinary education within the field of psychosocial health care and establish a National Graduate School in Innovative Psychosocial Health Care Research; (3) provide stimulating and challenging career opportunities for young researchers; (4) attract major external funding from the EU Framework Programs and Swedish research foundations; and (5) establish a Centre of Excellence, the Uppsala Care Centre (U-CARE Centre) for strong transdisciplinary research and research-based education within the field of psychosocial health care.

The original application described eHealth as a promising modality to be explored with the above goals in mind. Three randomized controlled trials (RCTs) evaluating Internet-supported interventions in the areas of pediatric oncology, adult oncology, and cardiology were already outlined in the application.

### Objective

The aim of this article was to describe challenges when establishing a publicly funded multidisciplinary eHealth research environment. The challenges encountered were (1) general challenges for developing new research programs such as the political environment, communication strategies and research relevance; and (2) challenges specific to eHealth and multidisciplinary programs such as software development and dealing with research cultures from several disciplines.

## Methods

### Design

The case for this study is the aforementioned U-CARE program. Nota bene, this paper is not an evaluation of the project’s main aims. Instead, it is a qualitative description of the challenges faced during the development process. Goal attainment for the project will only be briefly described to serve as a contextual frame of reference for the challenges described.

### Data Collection and Analysis

All senior and junior researchers working with the U-CARE program until 2014, as well as two of the project’s scientific advisors were invited to work with this paper. All besides one accepted the invitation and are consequently coauthors of this paper. Data were extracted from meeting protocols from the study coordination group, the program executive committee, the yearly meetings with the international Scientific Advisory Board (SAB), evaluation reports to funding agencies and the University administration, and from reflecting on own experiences. First, all coauthors working with the U-CARE program individually identified challenges in the documentation and wrote a summary of the key issues. Thereafter, the authors met and made a preliminary categorization of the identified challenges. At a second meeting, content, boundaries, and categorization of preliminary challenges were discussed and consensus was reached. Subsequently, the two first authors scrutinized the key issues, separating challenges general to developing research programs and challenges specific to multidisciplinary eHealth.

## Results and Discussion

### The Challenges

In this section, we describe the identified challenges and, in some cases, how they were handled. First, 3 themes with general challenges faced when setting up the program are presented. Thereafter, challenges more specific to eHealth and the multidisciplinary nature of the program are described, organized into 3 themes. A summary of the challenges can be found in Table 1.

### Organization

The U-CARE program was organized at the Disciplinary Domain of Medicine and Pharmacy at Uppsala University, above department level and was hosted by a relatively small existing research group at the Department of Public Health and Caring Sciences. It also included coworkers from other research groups, departments, and universities than from the host research group. A completely new organization was required for the strategic environment according to instructions from the university management matching the requirements from the funding agency while the program was part of the department’s organization. This meant that the coworkers had obligations both to their respective departments as employees, PhD students, teachers, or researchers, and to the new overarching organization. Sometimes, there were dual instructions. For example in U-CARE, the executive committee is responsible for the sanctioning of the PhD student’s study plans and for reviewing their progress. This resulted in redundancy as the departments already had detailed routines for this. An overflow of administrative duties may reduce academic output for example in terms of publications.

**Figure 1:**
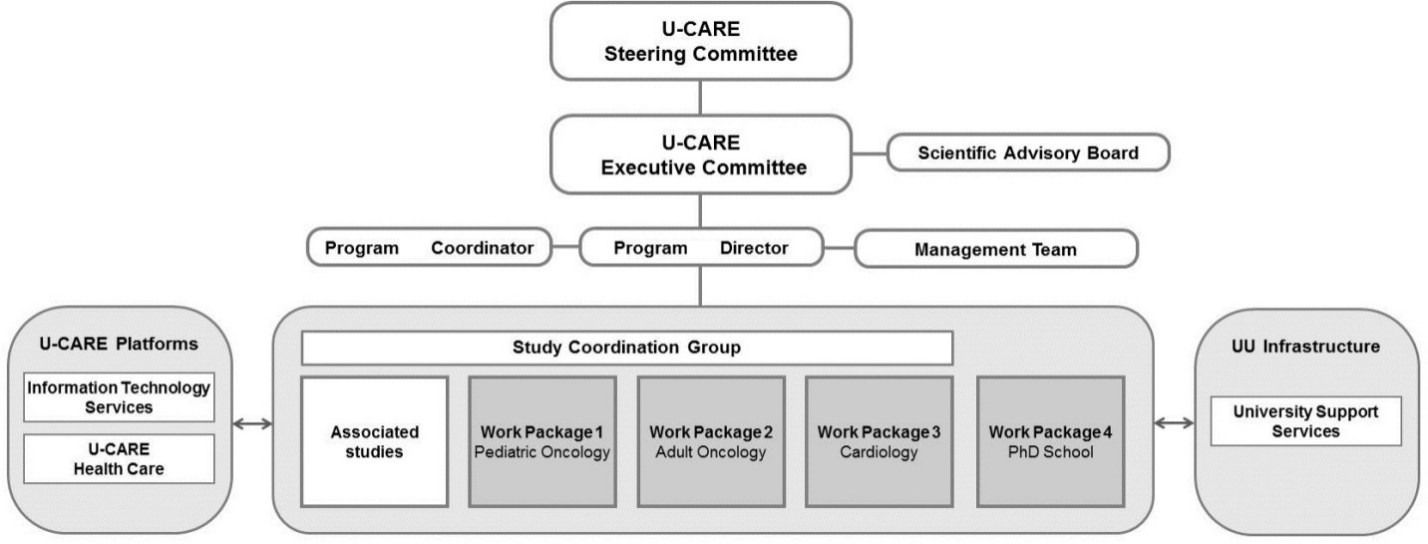
Organization of U-CARE.

Figure 1 illustrates the organization of the U-CARE program. The steering committee, executive committee, and SAB included members with expertise in caring science, clinical psychology, information systems, and health economics to facilitate the multidisciplinary work in the U-CARE program. The management team consisted of the Program Director, Program Coordinator, Information Technology Coordinator, and Research Coordinator. Work packages were organized around the RCTs that were already outlined in the application. These work packages as well as the associated studies (see below) were represented in the study coordination group meetings, which aimed at communicating the progress of each study and enabling synergy effects.

Studies initiated by other research groups were associated to U-CARE in order to benefit from the U-CARE portal. A more business-like practice emerged over time, where the associated studies became clients that purchased software as a service from U-CARE. The organization thus had to develop new knowledge and setup processes to act effectively as a service provider.

**Table 1:** Challenges in establishing a multidisciplinary research program on eHealth research.

General or specific to multidisciplinary eHealth projects	Themes	Challenges
**General**	Organization	1. The appropriate organization challenge 2. The strategic support challenge 3. The responsive organization challenge 4. The continuity of productivity challenge
	Communication	5. The internal communication and documentation challenge 6. The external communication challenge
	Implementation	7. The material sharing challenge 8. The stakeholder involvement challenge 9. The public involvement challenge
**Specific**	Legislation	10. The professor privilege challenge 11. The competing legislation challenge
	Software development	12. The mutual understanding of software requirements challenge 13. The software development documentation challenge
	Multidisciplinarity	14. The discipline openness challenge 15. The challenge of a shared theoretical framework

The appropriate organization challenge (#1) was to make the U-CARE program fit requirements from both the host university department and the funding agency as efficient as possible and with the least possible redundancy. The identification of the redundant or otherwise noneffective processes and routines was the key to necessary changes or adjustments. Our recommendation is that clarifications of responsibilities to the program and the host department should be made as soon as possible. One intention of the strategic research area initiative was to provide long-term funding to increase stability of the programs and relieve them from some of the demands normally put when applying for short-term project funding. Furthermore, the administration of a large program is demanding and time-consuming especially in the upstart phase. To facilitate the process, a program coordinator was hired at inception to support the program director. As the organization grew, responsibilities were delegated and duties were distributed within the group. Establishing a new research program is a long-term investment and takes time and effort to build the most efficient organization and administrative routines.

The strategic support challenge (#2) was to attract support from the head of the department, the vice-chancellor, and officials at university management level. A research group, receiving a large multidisciplinary grant, needs support on a strategic level to establish career opportunities as well as provide appropriate infrastructure aligned with the program. Such support is necessary to establish a successful and sustainable program that does not come easy. When this was being written, the program has moved to another host department in an attempt to improve the organizational conditions for the program.

A large program needs to be organized rigorously to meet the high demands on quality and transparency from both the funder and the scientific society. The responsive organization challenge (#3) was to make the organization flexible and responsive to new ideas and a changing environment while keeping scientific rigor and quality. This challenge was met in a very general way by putting efforts into never loosing focus on the overarching goals of the project, even when administrative short-term goals were calling for attention. This is not a challenge that was solved once and for all. As is the case with many challenges, facing them is a continuing process.

The continuity of productivity challenge (#4) was to keep an even pace of production that is, publishing articles while starting up large, time-consuming studies. The U-CARE portal was developed from scratch and all the large projects started up in parallel. The way we handled this challenge was by starting additional short-term projects to get a workable mix. This meant that the original RCTs were complemented with other types of studies. For example, brief intervention studies with student participants, qualitative interview-based studies, feasibility and pilot studies, registry-based studies, and studies describing the project development processes including trial protocols were accomplished (eg, [8-14]). It is important for a research organization’s output to have nonsynchronized and timely overlapping projects.

### Communication

Internal and external communication is a key factor for efficiency and goal attainment. Accordingly, much time and effort was devoted to establishing a clear intra- as well as an interorganizational communication strategy. The strategy described different information systems and communication channels for different purposes. The strategy serves as the blueprint for communication with the public, stakeholders, and between colleagues. The project’s external and internal website, emails, shared server folders, the U-CARE portal itself, Web-based meeting systems, cloud technologies, and face-to-face meetings were important communication channels. Although this is an eHealth program, personal meetings ranging from regular working group meetings to international scientific conferences have been very important. The internal communication and documentation challenge (#5) was to implement an efficient strategy for internal communication aiming at making communication, information sharing, and documentation part of employees’ daily routines and to ensure that all ongoing research and educational activities were documented properly. This documentation was the basis for the yearly reports of progress and productivity to funding sources. To meet these challenges, a combination of a general strategy building commitment to the project and a more specific strategy with frequent reminders and easy-to-use-templates to fill in was used.

The external communication challenge (#6) was to establish efficient external communication. One key task was to set up the U-CARE website. It allowed for effective and transparent external information sharing. The website served several of the defined communication purposes as it provided continuously updated and easily accessible information regarding main deliverables, ongoing studies, the organization structure of U-CARE, meeting protocols from the decision-making bodies, and policies, and so on. News was also distributed using Twitter with links to the U-CARE website for more information. In line with the university’s third assignment, that is, to inform citizens, efforts were made to present the work to the public as well as politicians and health care officials. This was done by contacting the media, by participating in meetings arranged by nonprofit and patient organizations, and by inviting to a seminar at a popular societal and political yearly national event in Sweden (Almedalen) where many different stakeholders gathered.

### Implementation

An important goal from the start for U-CARE was that any successful intervention should be implementable in regular care. Implementation of an intervention must depend on its efficacy and effectiveness, which is currently being evaluated in the RCTs. Thus, the implementation process cannot start until it is known whether an intervention is worth implementing, as the ideal in health care is to implement only evidence-based interventions. When it comes to eHealth, this creates a special difficulty as evidence takes a long time whereas the technological development is fast. At the time when a sufficient level of evidence is reached, the technology might be outdated. This may be a future challenge for U-CARE.

The idea of implementability has influenced the design of the U-CARE program from the start. For example, the recruitment of participants to the studies is done consecutively in clinical settings when possible, which is actually rare in the Internet therapy literature [15]. In addition, the material created for the portal was licensed to open access as per the most liberal Creative Commons license at the time, CC BY, where anyone is allowed to use and alter the material as long as it is attributed to the original author. It may seem uncontroversial but there are some potential negative side effects from a business perspective. The material sharing challenge (#7) is how to share the material for free without creating unfair competition or to limit the commercial interest. This challenge has not yet specifically been met in other ways than that it is acknowledged as a potential risk. A similar challenge applies to the use of open source code. In this case, we chose not to use open source, at least for now.

Newly developed eHealth technology has often had difficulties in reaching and being implemented in health care practices. This has been analyzed by, among others, van Gemert-Pijnen and colleagues [16]. They found that eHealth development often disregards the relationship among technology, human characteristics, and the socioeconomic environment. This led them to develop and suggest the CEHRES roadmap for development of eHealth technology, which emphasizes the interaction with the different stakeholders [16]. Although the CEHRES roadmap was not published when the U-CARE program started, several stakeholders were identified and approached early. The stakeholder involvement challenge (#8) was to identify and involve appropriate stakeholders. Researchers and clinicians such as psychologists and nurses have had a large input on the development of the program and the interventions. Other stakeholders who have not yet been involved are the future service providers, such as commercial or public clinics, who may deliver the interventions in regular care. For future implementation, it is also crucial to inform and engage a larger group of stakeholders such as politicians and health care officials early. Their engagement and knowledge is important for collaboration, implementation, and ultimately, value for the society.

The importance of interacting with the end-users in every part of the research process has become clear over the evolution of the program. Patient and public involvement (PPI) in research is recognized as an important strategy to ensure relevance and legitimacy of research activities and findings [17]. The involvement of patients in U-CARE aimed to ensure the clinical relevance and legitimacy of the interventions, the outcome measures, and the user interface of the U-CARE portal. In addition, after the interventions were prepared, patients have remained a valuable source of knowledge to improve the research projects. Involving patients in research activities was a novel approach to most researchers in U-CARE, and this work has led to an awareness of the complexity and costs of patient involvement as well as the benefits [18]. The public involvement challenge (#9) was to allocate time and the resources to do this work. It is not reasonable to expect contributions over several years from patient representatives without any compensation. Such resources were not planned for at an early stage and although PPI has been applied during the course of the program, greater efforts could have been made to take advantage of it. Ideally, a program of this size should have an assigned person with expertise in PPI to plan, educate, and allocate resources. Efforts were also made to change the researchers’ view of patients from study objects to research partners. To enhance this work, it would be a good idea to educate both patient representatives and researchers on how to collaborate in research.

### Legislation

A number of legislative challenges have arisen during the program, mainly concerning research, health care, and copyright.

The Swedish copyright legislation differs from other countries’ when it comes to innovations conducted by a university employee. According to the unique Swedish professor’s privilege, teachers and researchers have the rights to the intellectual property that originate from research, development, and innovation activities. This created the professor privilege challenge (#10), as it means that the university as an employer has neither rights nor direct incentives to manage these kinds of innovations [19]. It is up to the respective owner of the intellectual property, which in U-CARE’s case includes many persons, to take care of how the end product should be handled (eg, advertised and maintained). With the intention to benefit as many as possible, an important ambition of the U-CARE-program was to share material with others. This has required contracts with U-CARE staff, to license the material according to the aforementioned liberal CC BY-license. This has been done to remove some of the legislative barriers and facilitate future implementation.

At the onset of the program, the focus was on legal requirements regarding research ethics and personal integrity, which U-CARE researchers had previous experience with. The setting for the U-CARE program is academic, not clinical. When setting up the studies and routines, it became evident that no caregiver system was in place, which is required for psychological treatment in Sweden. After several discussions with the university management and National Board of Health and Welfare, it was decided to create a new caregiver organization, independent from the hospital or other clinical entities, at Uppsala University specifically for the research within U-CARE, a solution that hitherto has been very uncommon. As a result, the legal office at the university was obliged to support this new activity.

Legislation and practice around health care and research are not always in concordance. The competing legislation challenge (#11) has been to navigate these two legal systems. In research, for example, data should be as transparent as possible to other researchers (eg, Open data); whereas in health care, patients’ integrity is of central importance. Another example is that in research the ideal is that the therapists are as blind as possible to the participants, for example, the baseline measures, whereas in medical practice it is essential to know details about the patient and to keep a medical record. This challenge is also a consequence of the double role that is common in clinical research, where the researchers are also therapists. Relating to this there is also an eHealth legislation lag. eHealth and related research has developed fast and the legislation is often not updated on this area, at least not in Sweden. This requires adaption to existing but not always appropriate Health and Medical Services legislation. For example, the distinction between research material and medical record information is not always clear. In two of the ongoing U-CARE studies, only participants who scored above a certain threshold level of anxiety and depression are offered to take part in an intervention after randomization. In this situation, it is not clear who are U-CARE’s patients from a health care perspective and for whom a medical record is mandatory—For all the persons screened; for those randomized to treatment; or for all scoring above the threshold? On top of this, there are several new data safety issues that call for updated regulation, for example, cloud technologies.

### Software Development

At the inception of U-CARE, the market was reviewed for software that could support the interventions and research for the planned studies in an appropriate way. No such software was found on the Swedish market. Hence, a strategic decision was made to collaborate with the information systems discipline in the process of developing the software program. Information systems researchers were engaged to participate both as researchers and as software developers. The decision meant that no external software consultants were engaged to help build the software. The information systems research focus was initially explorative and its research aim was somewhat unclear, but it became more focused with time.

The research in U-CARE largely depended on the U-CARE portal, which was adapted to both research and treatment needs. During the development phase, all parties underestimated the complexity of the software to be designed. Complexity was caused by a series of requirements, including (but not limited to) that the software:

- should be flexible so that it would suit the diverging needs of the different studies
- should be easy-to-use for people with diverging computer skills, provide a rich user experience, and feel attractive and modern
- should stimulate interaction between users, primarily among participants and between therapists and participants
- had to comply with current security standards and privacy regulations according to Swedish legislation
- could monitor study characteristics and provide updated study progress reports on recruitment and other parameters

The increasing complexity caused by a continuous flow of new requirements and applications from the clinical trial researchers led to considerable postponement of the launching of the U-CARE portal, which in turn affected the starting point of the planned studies.

When professionals with different backgrounds, for example, with regard to profession and academic discipline work together, there are communication challenges. In relation to software development, this is a well-known phenomenon. We had to make sure that the expressed and perceived expectations with respect to the system were in agreement. Here, we call this the mutual understanding of software requirements challenge (#12). It is hard to make demands within a field, for example, programming of software functions, in which you are not oriented. In the same way, it is difficult to respond to a demand that comes from an environment, for example, the clinical, that is unfamiliar. This challenge was met by adopting an iterative, agile, software development process and by weekly meetings between the developers or information systems researchers and the psychologists or clinical researchers [20]. The agile approach emphasizes continuous interaction between stakeholders and adaptation to changing customer requirements, in contrast to traditional plan-driven approaches to the, so-called, software development life cycle.

An additional software development documentation challenge (#13) was the need for continuous documentation of the development of the portal. New functionalities and complex configurations were added continuously as the portal developed, and with increasing time pressure, documentation was to some extent down prioritized. The lack of such documentation increases the risk to become dependent on certain individual’s inherent knowledge of the code. Routines for documentation that were set up were not always adhered to. These routines were successively improved. It is of uttermost importance to have well-functioning and easy-to-use routines and management systems to maintain them.

### Multidisciplinary Research

The development of multidisciplinary collaborations was included in U-CARE’s fundamentals. For example, cost-benefit analyses were included in the original RCTs from the very start. This led to cooperation with researchers from health economics. As aforementioned, it was also decided early in the process that the development of the Internet portal was to be included as an integral part of the U-CARE program in contrast to using an existing portal or to work with IT-developers as consultants. Consequently, the technological development process became a research topic in itself mainly from an information systems perspective involving researchers from the informatics discipline. It was a general expectation that disciplines working together would be a success factor for dealing with complex research questions, for example, at cross-borders between the individual, the society, and a technological environment. It was also anticipated that down the road toward true interdisciplinarity, several obstacles would have to be dealt with [21]. At the outset, little was known about each other’s cultures, structures, terminology, and science. Hence, it can be described as a discipline openness challenge (#14) to adjust to, be open to each other’s qualities and expectations, and allocate resources in order to integrate our knowledge to reach new results. It proved to entail a greater intellectual challenge than was imagined. The initial tendency was to accumulate rather than integrate (eg, the interests of several research areas resulted in a larger number of questionnaires), with the risk of not satisfying any of the involved disciplines. Alternatively, making one discipline primary with priority to outline the research design would lead to others being secondary, marginalized, and unsatisfied.

The multidisciplinary ambition also resulted in higher demands on coordination and communication as the group became more diverse regarding professional and educational background as well as in research objectives. Collaboration was hampered by organizational differences with different systems in different departments, particularly for PhD students who had their project in one discipline or department and the main supervisor in another. Different cultures regarding PhD studies also made the integration more complicated. For an individual PhD student, the primary goal is to meet the requirements of the own discipline. Differences between disciplines regarding the various aspects of publication (type of publication, number of authors, author order, lack of interdisciplinary journals, etc) was another example of a cultural difference that was not fully anticipated beforehand.

Discussions regarding theoretical basis and research objectives have been ongoing and differences in scientific philosophy have been revealed along the way. The expectation that different scientific approaches are enriching has been confirmed. However, too large differences may also obstruct communication. The starting point for U-CARE was societal and clinical relevance rather than theoretical curiosity. The idea of theoretical framing and potential theoretical contributions emerged over time. However, given the large number of ongoing studies and the various backgrounds of the involved researchers, different subgroups relate their work to theory in different ways, without an agreed-upon shared theoretical framework. To bridge this gap was the challenge of a shared theoretical framework (#15). Several candidate theories to frame the studies were proposed including, but not limited to, learning theory, which is the base of cognitive behavioral therapy, and socio-materiality [22,23]. In addition, pragmatism has served as a philosophical starting point to guide and frame strategic research choices. We also applied more nontheoretical frameworks, for example, the British Medical Research Council’s framework for development of complex interventions [24]. An implication of the lack of coordination in this respect may be a reduced long-term opportunity for theoretical contributions related to the design and application of technology for online psychosocial support. Moreover, the lack of an agreed-upon labeling of ongoing work may also lead to less-efficient communication of results to both academia and practice. Although letting each researcher frame results in a way that makes sense in his or her specific field may be effective and flexible, coordination is needed to make a true multidisciplinary contribution.

### U-CARE’s Accomplishments

So, was U-CARE a successful program? By the end of 2014, the following had been accomplished regarding the goals set in 2009 (see U-CARE’s project goals above).

Regarding establishing a research platform, the most significant output since the inception of the U-CARE program has been building an Internet portal, that is, the U-CARE portal, a generic and flexible Internet portal that supports collection of data and provision of psychosocial care and psychological treatment. Web-based psychosocial and psychological self-help programs have been developed for pediatric oncology, adult oncology, and cardiology [8,9,11,14]. Within the U-CARE program an infrastructure, that is, logistics, structures, and policies, for delivering care and psychological treatment online has been created. A number of research groups have been associated to the U-CARE program in order to use the U-CARE portal and the self-help programs developed within U-CARE. The associated groups contribute with Web-based open access self-help material for groups such as pregnant women with fear of giving birth; women who experience post-traumatic stress after a difficult delivery or abortion; persons with risk for recurrence of depression; children who experience pain due to mucositis, and so on. By the end of 2014, 6 studies had started in the portal, all targeting Swedish participants. In the first goal, (1) international competitiveness and innovation were stressed on, but these aspects are difficult to evaluate factually at the moment. (2) A research-based multidisciplinary education within the field of psychosocial health care and a national graduate school in innovative psychosocial health care research has been organized. Eight PhD students started the graduate school in 2011 or 2012. Three multidisciplinary courses on eHealth-related topics have run between 2012 and 2014. (3) Regarding career opportunities, the following figures indicate a positive result. In 2009, the research group where U-CARE is hosted consisted of 6 persons. By the end of 2010, the group already consisted of 25 persons, and the size of the research group has been relatively stable throughout 2014. In addition, approximately 25 persons with positions at other departments at Uppsala University and 10 persons at other Swedish universities are involved in the U-CARE program. In total, 13 PhD students are involved in the U-CARE program. Four post-doctoral positions have become permanent researcher positions. (4) When it comes to attracting major external funding from major Swedish research foundations, the U-CARE program has been successful. In total, 19.354.900 SEK (approximately €2.134.000) have been received in addition to the original funding for research activities between 2011 and 2014. At the moment, 29.930.000 SEK (approximately €3.300.000) has been granted for 2015-2018. However, no funding has been applied for and thus not been received from the EU framework program. (5) Originally, there was a plan for a center of excellence organized independently of the existing university departments. However, these plans were changed already at the inception because of organizational reasons. U-CARE has since inception throughout 2016 been hosted at the Department of Public Health and Caring Sciences, group: Clinical Psychology in Healthcare, Uppsala University.

In May 2015, the Swedish Research Council published a comprehensive evaluation of all the 43 programs in the Strategic Research Area-Initiative. All programs were evaluated by an expert panel regarding, among other things, research output, collaborations, and management. The conclusion was that U-CARE has developed satisfactorily in many aspects, especially when it comes to management, association with other national research groups, and for effectively starting up a new organization within a new field of research. The main challenges for the future pointed out by the experts were research output, that is, scientific publications, international collaborations, impact on business, and contribution to undergraduate education [25].

### Conclusions

Establishing a publicly funded multidisciplinary eHealth research environment is a challenging endeavor. Although several challenges were met during the development of the U-CARE program, several accomplishments were also made. Some of which could not have been made if it weren’t for this new long-term research environment. By sharing our experience, we hope to help other research groups embarking on a similar journey to be prepared for some of the challenges they are likely to meet on their way. We do anticipate future challenges within these themes that are not described in this paper. This involves implementation in care if the interventions prove to be efficient, continuous adjustments to new technologies, and legislation. By reflecting on our previous challenges, we are better armed to face the challenges of the future.

For some challenges, we provide a solution that worked for us. For other challenges, we have suggestions that we have not fully implemented. We recommend our readers to reflect on how to tackle the challenges and anticipate that being prepared will help to tackle these challenges in a more efficient way.

## Abbreviations

PPI: patient and public involvement
RCT: randomized controlled trial
SAB: Scientific Advisory Board
U-CARE: Uppsala University Psychosocial Care Program
